# A transition zone complex of ciliopathy proteins regulates ciliary composition

**DOI:** 10.1186/2046-2530-1-S1-O16

**Published:** 2012-11-16

**Authors:** JF Reiter, F Garcia-Gonzalo, K Corbit, W Dowdle, L Yee

**Affiliations:** 1 Department of Biochemistry and Biophysics, Cardiovascular Research Institute, University of California, San Francisco, USA

## 

We have identified a complex of proteins that form part of the transition zone, a region at the base of the cilium. This complex includes the three members of the Tectonic family, extracytosolic glycoproteins that interact with transmembrane components of the transition zone such as Tmem67, Tmem216, and Tmem231. These transmembrane proteins connect to an intracellular transition zone complex comprised of many known Joubert- and Meckel-associated proteins including Cc2d2a, B9d1, B9d2, Mks1. Loss of components of this transition zone complex in mice compromise ciliogenesis in some tissues, and deregulate ciliary protein composition in others. In particular, the ciliary localization of Smoothened (Smo), a central component of the Hedgehog pathway, depends on this complex. As Smo functions at the cilium, many mouse transition zone mutants show deregulation of Hh signaling, resulting in ventralization of the neural tube and polydactyly. Defining the components of the transition zone has led to the identification of additional genes underlying Joubert and Meckel syndromes including Tctn1, Tctn2 and B9d2. We hypothesize that Joubert and Meckel syndromes are caused by transition zone dysfunction that disrupts intercellular signaling, leading to developmental defects.

